# Symptomatic Lumbar Discal Cyst: A Rare Entity that Can Mimic Other Lumbar Cystic Lesions

**DOI:** 10.7759/cureus.5453

**Published:** 2019-08-21

**Authors:** Zaid Aljuboori, Thomas Altstadt

**Affiliations:** 1 Neurosurgery, University of Louisville School of Medicine, Louisville, USA

**Keywords:** lumbar, disc, cyst, radiculopathy

## Abstract

Lumbar discal cyst (LDC) is a rare clinical entity with unclear etiology. Three theories have been proposed to describe the pathogenesis of this condition: (i) a reaction to spinal epidural hematoma; (ii) a pseudomembrane formation that follows the focal annular tear and disc degeneration; and (iii) an inflammatory reaction to the herniated disc fragment. It usually presents with radicular symptoms. Radiographically, LDC can mimic other cystic lesions of the lumbar spine. Imaging nuances such as scalloping of the vertebral body, contrast filling with discography, and MRI signal intensity on different sequences can help to establish a diagnosis of LDC. However, there are no clear guidelines on the best treatment approach. Several treatment options have been prescribed to treat LDC with good outcomes. Here we describe a case of LDC that presented with left-sided radicular symptoms for several months and had not undergone any initial conservative management. The patient was treated successfully with microscopic resection of the cyst with complete resolution of the symptoms.

## Introduction

Lumbosacral radiculopathy is a clinical pain syndrome caused by irritation, tension on, or compression of the spinal nerves. Different etiologies can cause this condition, such as disc herniation, facet hypertrophy, ligamentum flavum hypertrophy, and presence of compressive cysts [1, 2]. It usually presents as low-back pain that radiates along the dermatomal distribution of the involved spinal nerve. Patients may also experience paresthesia, hypoesthesia, muscle weakness, or hyporeflexia [1, 2].

There are different types of cysts that can exist extradurally in the lumbar spine, specifically in the spinal canal and neuroforamen. Common examples include synovial cysts, ganglion cysts, and Tarlov cysts. Patients with these lesions can present with lumbar radiculopathy [3-6].

Lumbar discal cyst (LDC) is a rare clinical entity with unknown etiology that has only been recently diagnosed. It can present as lumbar radiculopathy [7]. Clinically and on imaging, it can be easily confused with other types of cysts of different etiology, natural history, and treatment paradigm. Here we describe a case of symptomatic LDC that was treated microsurgically. We also present the hypotheses regarding the pathophysiology of this condition and imaging nuances that can help to differentiate LDC from other cystic lesions.

## Case presentation

**Figure 1:**
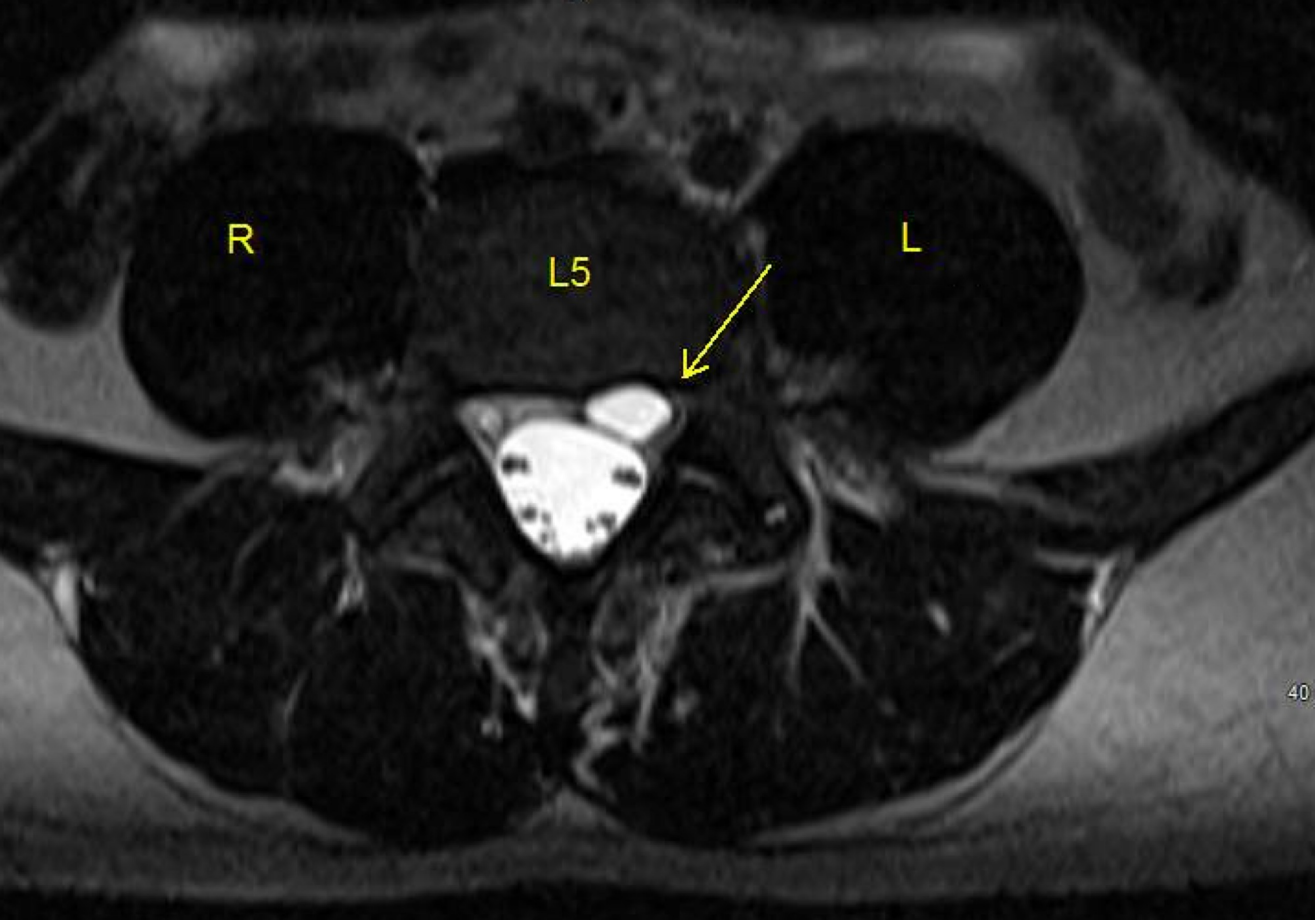
MRI T2 sequence of lumbar spine (axial view). Shows a left-sided hyperintense cystic lesion behind the L4 vertebral body (arrow).

**Figure 2:**
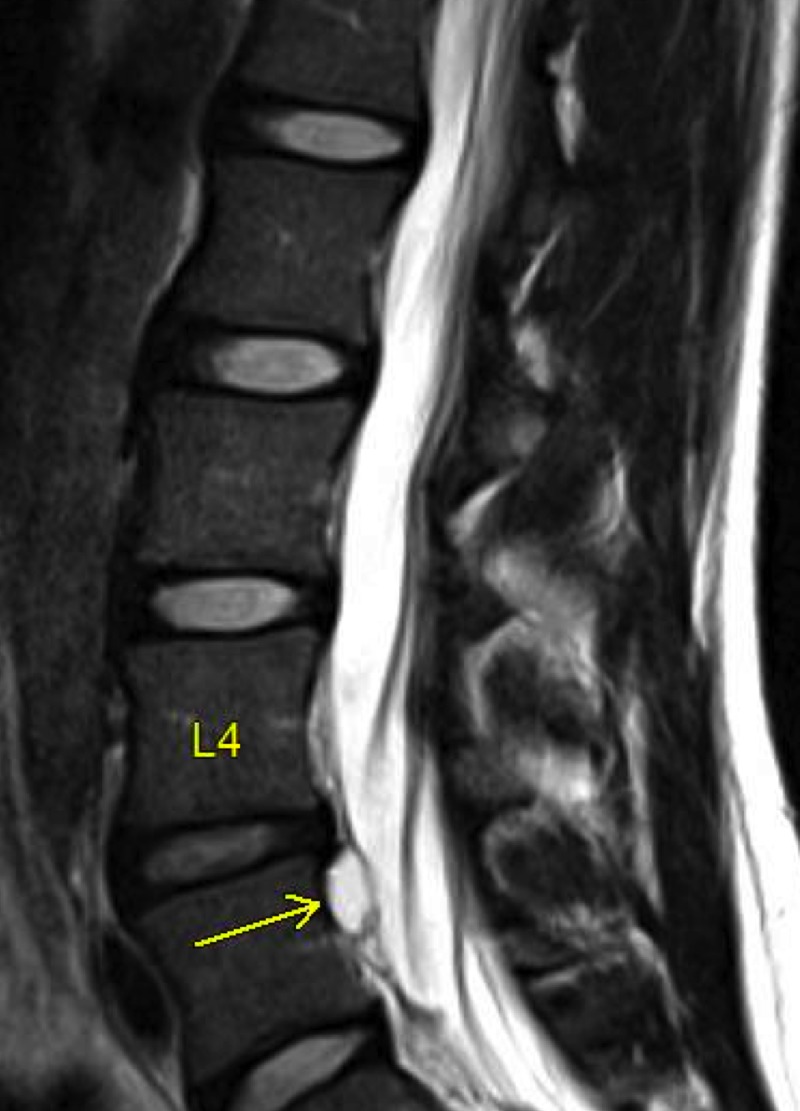
MRI T2 sequence of lumbar spine (sagittal view). Shows a hyperintense cystic lesion behind the L4 vertebral body (arrow).

The patient was a 24-year-old female who presented with six-month-old low-back and left-leg pain. The pain had started suddenly when she once bent over while doing laundry. She described the pain as a sharp, shooting one that had started in the lower back and travelled along the lateral aspect of the thigh and terminated at the knee. Initially, the pain was treated empirically with nonsteroidal anti-inflammatory drugs and intramuscular steroid injection, which resulted in short-lasting improvement. Later on, the patient started experiencing paraesthesia of the lateral aspect of the left thigh. Due to new sensory changes and the failure of conservative therapy, the patient underwent an MRI of the lumbar spine six months after the initial presentation. The MRI showed a left-sided extradural cyst at the level of 4th/5th lumbar vertebra (L4/5). The cyst was hypointense and hyperintense on T1- and T2-weighted images, respectively (Figures 1-2).

**Figure 3:**
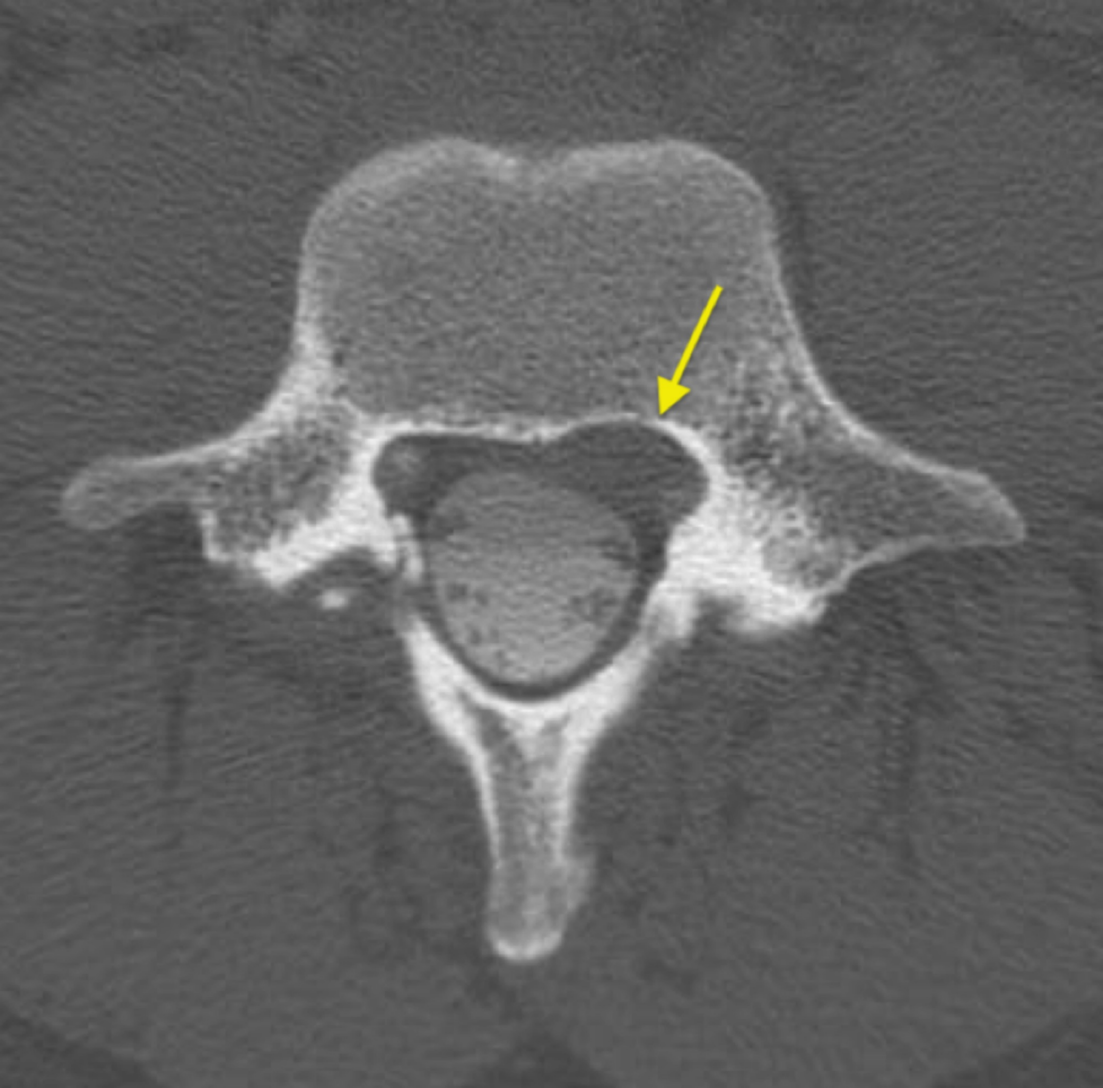
CT myelogram of the lumbar spine. Shows a left-sided cystic lesion with scalloping of the posterior vertebral body (arrow) with no contrast filling.

The patient also underwent a CT myelogram, which showed scalloping of the posterior vertebral body of L4 and no filling of the cyst with contrast (Figure 3).

**Figure 4:**
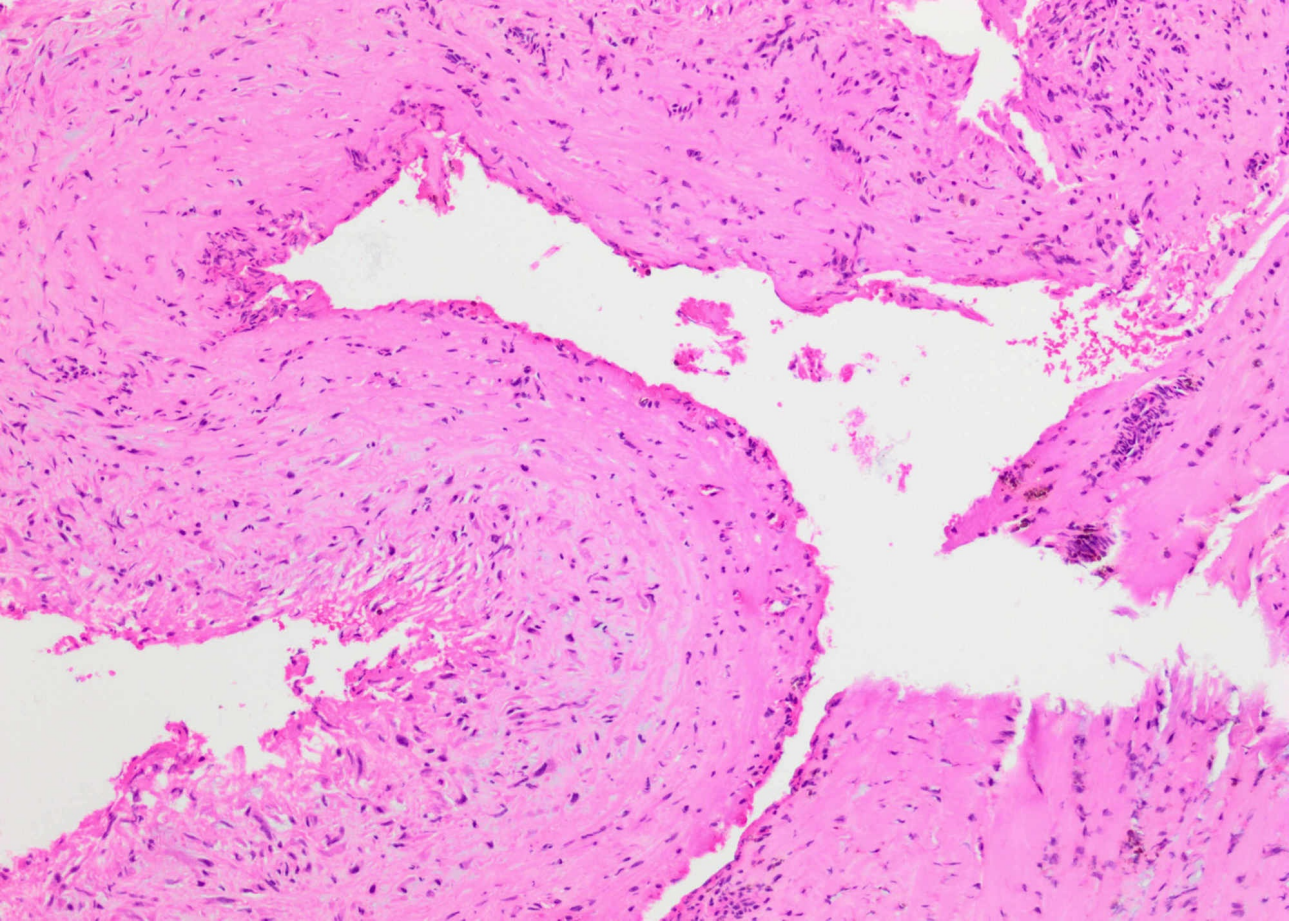
H&E preparation of the cyst wall (10x). Fibrosis and absence of epithelial lining of cyst wall.

**Figure 5:**
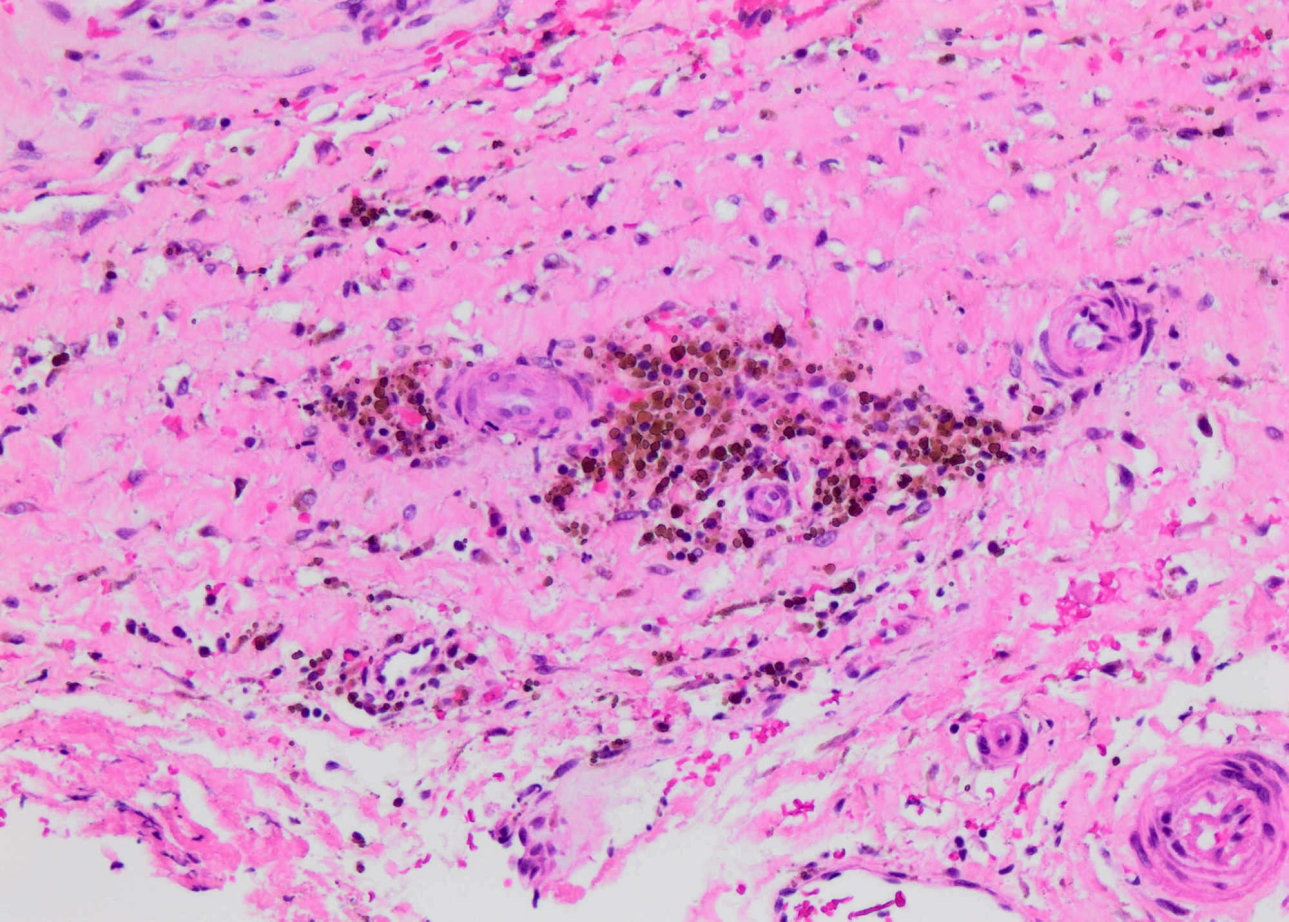
H&E preparation of the cyst wall (20x). Hemosiderin deposition present within the cyst wall.

A physical examination of the patient showed weakness of the left extensor hallucis longus (4+/5 motor power). The patient then underwent a minimally invasive microscopic resection of the cyst. Postoperatively, she noticed immediate improvement in her symptoms. Pathological analysis demonstrated fibrosis and hemosiderin deposition within the cyst wall and absence of epithelial lining (Figures 4-5). At a follow-up visit after six weeks, her pain was found to be completely resolved and she had normal neurological exam. 

## Discussion

LDC is a newly diagnosed pathology generally involving the lumbar spine [7]. The condition's exact etiology is unknown, but there are three hypotheses described in the literature regarding its pathogenesis [7-9]. The epidural hematoma hypothesis states that an initial hemorrhage occurs from the epidural venous plexus followed by a reactive inflammatory response, which leads to the formation of the cyst. This hypothesis is supported by the presence of hemosiderin deposits within the cyst wall [7]. The reactive pseudomembrane theory described by Kono et al. argues that the process starts with an initial focal degeneration of the posterior disc wall and fluid formation. This is followed by the leaking of fluid into the epidural space, which mounts an inflammatory reaction and the formation of a pseudomembrane around it [9]. Histological findings that support this hypothesis are the presence of the fibrous connective tissue, imaging finding of an annular fissure, and a communication between the intervertebral disc and the cyst. The third hypothesis is a variant of pseudomembrane theory. This states that the cyst forms as an end result of focal disc herniation and resorption by inflammatory response [8]. Histological findings in favor of this hypothesis are the presence of the disc material in the cyst and the presence of macrophages and fibroblast in the cyst wall. 

Imaging nuances such as the location of the cyst, bony changes on CT, contrast filling with discography or myelography, and MRI characteristics can be useful to differentiate between different types of cysts. LDC is almost always located behind the posterior vertebral body, which is also a typical location for herniated discs [7]. Ganglion and synovial cysts are usually located in close proximity to the lateral aspect of the lamina and facet joint [10]. Tarlov (perineural) cysts are generally located anterolaterally within or in proximity to the neuroforamen [11, 12]. On CT, LDC appears as a hypodense mass that may cause scalloping of the posterior vertebral body. It fills with contrast if CT is done after discography [8, 13]. On CT myelogram, only Tarlov cyst usually fills with contrast [14]. On MRI, LDC appears as hypointense on T1-weighted image and hyperintense on T2-weighted image, and enhances homogenously after gadolinium [13]. Synovial and ganglion cysts have similar MRI characteristics to LDC [5]. Tarlov cyst appears as hypointense on T1-weighted image and hyperintense on T2-weighted image, with no enhancement after gadolinium [15].

There are no guidelines regarding the best treatment option for LDC. Most evidences are in the form of case reports or series. Different treatment approaches have been prescribed, including observation, percutaneous needle aspiration, endoscopic resection, microscopic resection, and laser ablation [16-20]. Prasad et al. reported a case of symptomatic LDC that was treated conservatively with epidural steroid injection. A follow-up MRI after five months showed complete regression of the cyst with resolution of the patient’s symptoms [16]. Jha et al. reported a symptomatic LDC that failed six months of conservative therapy and eventually needed surgical intervention. They used the endoscopic transforaminal route for the removal of the disc fragment and the associated discal cyst, which resulted in resolution of the patient’s symptoms and the complete regression of LDC on MRI two months after the surgery [17]. A possible explanation for the failure of conservative therapy in the case reported by Jha et al. is the presence of a disc fragment within the cyst. Since disk degeneration is a proposed mechanism for the development of discal cysts, the persistent presence of the disc fragment will continue to trigger inflammatory response. In addition, Prasad et al. used epidural steroid injection, which was not used in the Jha et al. case. 

Yu et al. reported a case of symptomatic LDC that developed shortly after the surgical resection of a herniated disc. They treated it with fluoroscopy-guided needle aspiration, which resulted in resolution of the patient’s symptoms [20]. Kim et al. used YAG laser to treat a group of patients with LDC that had failed short-term conservative therapy. The average postoperative follow-up period was after 12 months. They reported resolution of the symptoms with regression of LDC on the follow-up MRI [18]. The reports discussed in this article demonstrate some variability in the development, presentation, and response to different treatment strategies of LDC. This attests to the lack of a complete understanding of this disease process. In addition, there are no data in the literature regarding the recurrence of this pathology after surgical treatment. 

Despite the lack of any high-level evidence for the management of LDC, a treatment approach that begins with a conservative therapy (i.e., epidural steroid injections; oral, nonsteroidal anti-inflammatory medications), with surgery reserved for patients who fail it, can be used to manage symptomatic LDC. Recognition of this clinical entity is of the utmost importance, since it is easily treatable with minimally invasive surgical approaches with excellent outcomes. In addition, it poses minimal surgical risks. 

## Conclusions

LDC is a rare clinical entity that has only been recently diagnosed. The exact pathogenesis of this condition is yet to be elucidated. If symptomatic, LDC usually presents with lumbar radiculopathy. On imaging, it can be confused with other cystic lesions of the lumbar spine. Several treatment options have been prescribed, with most resulting in the regression of the cyst and subsequent resolution of the symptoms. Initial conservative therapy, with surgery reserved for patients who fail it, can be used to manage this condition.
